# Characterization of AlgMsp, an Alginate Lyase from *Microbulbifer* sp. 6532A

**DOI:** 10.1371/journal.pone.0112939

**Published:** 2014-11-19

**Authors:** Steven M. Swift, Jeffrey W. Hudgens, Ryan D. Heselpoth, Patrick M. Bales, Daniel C. Nelson

**Affiliations:** 1 Institute for Bioscience and Biotechnology Research, University of Maryland, Rockville, Maryland, United States of America; 2 National Institute of Standards and Technology, Biomolecular Measurement Division, Gaithersburg, Maryland, United States of America; 3 Department of Veterinary Medicine, University of Maryland, College Park, Maryland, United States of America; NERC Centre for Ecology & Hydrology, United Kingdom

## Abstract

Alginate is a polysaccharide produced by certain seaweeds and bacteria that consists of mannuronic acid and guluronic acid residues. Seaweed alginate is used in food and industrial chemical processes, while the biosynthesis of bacterial alginate is associated with pathogenic *Pseudomonas aeruginosa.* Alginate lyases cleave this polysaccharide into short oligo-uronates and thus have the potential to be utilized for both industrial and medicinal applications. An alginate lyase gene, *algMsp*, from *Microbulbifer* sp. 6532A, was synthesized as an *E.coli* codon-optimized clone. The resulting 37 kDa recombinant protein, AlgMsp, was expressed, purified and characterized. The alginate lyase displayed highest activity at pH 8 and 0.2 M NaCl. Activity of the alginate lyase was greatest at 50°C; however the enzyme was not stable over time when incubated at 50°C. The alginate lyase was still highly active at 25°C and displayed little or no loss of activity after 24 hours at 25°C. The activity of AlgMsp was not dependent on the presence of divalent cations. Comparing activity of the lyase against polymannuronic acid and polyguluronic acid substrates showed a higher turnover rate for polymannuronic acid. However, AlgMSP exhibited greater catalytic efficiency with the polyguluronic acid substrate. Prolonged AlgMsp-mediated degradation of alginate produced dimer, trimer, tetramer, and pentamer oligo-uronates.

## Introduction

Alginate is a linear polysaccharide composed of two different uronic acids,β-D-mannuronic acid (M) and α-L-guluronic acid (G). These two uronic acids are present in alginate as polymannuronic acid blocks (polyM), polyguluronic acid blocks (polyG) and in mixed MG regions. Alginate is produced by seaweeds and by some *Pseudomonas* and *Azobacter* bacteria [1].

Alginate lyases are enzymes that degrade alginate by a β-elimination reaction that breaks the 1–4 O-linkage between the uronic acids in the linear polymer. This reaction results in the formation of a double bond between carbons 4 and 5 of the uronic acid that was linked at the 4^th^ carbon. Alginate lyases can be endolyases, cutting the polysaccharide internally, or exolyases, cutting at the end of the polysaccharide [2], [3]. Alginate lyases can have substrate preferences for polyM or polyG present in the alginate.

Brown seaweed is the source for commercial alginate, with the major seaweed species being from *Macrocystis*, *Laminaria*, and *Ascophyllum* [1]. Different seaweeds produce alginates with different ratios of M and G residues. Alginate extracted from seaweed is used in food as a thickening or gelling agent, as well as a stabilizer. Alginate hydrogels are used in some bandages for treating burns [4], and alginate hydrogel capsules can be used as a drug delivery vehicle [5], [6]. Alginate lyases are of interest for processing seaweed alginate for industrial, pharmaceutical, and biofuel applications [1], [7], [8].

Another source of alginate is bacteria. *Pseudomonas aeruginosa* is associated with lung infections in patients with cystic fibrosis [9]. *Pseudomonas* isolated from these patients typically have a mucoid phenotype [10] and secrete alginate [11]. Alginate secreted by *Pseudomonas* species is incorporated into an extracellular matrix called a biofilm. Beyond protecting bacteria from environmental stress, biofilms are also known to protect bacteria from antimicrobials, such as antibiotics [12]. Significantly, alginate lyases have been shown to improve the efficacy of antibiotics against *Pseudomonas aeruginosa* in biofilms [13], and are therefore of medical interest in treating lung infections from *Pseudomonas*.

Many alginate lyases have been isolated from organisms that feed on seaweed, mostly marine bacteria and also from sea snail gut [3]. Some bacteria have several alginate lyase genes. A prime example of which is *Saccharophagus degradans* 2–40, which has 13 predicted alginate lyase genes [14]. Having multiple alginolytic enzymes with different properties, for instance secreted versus attached to the cell exterior or preferential degradation of polyM versus polyG regions in alginate, increases the ability of marine bacteria to utilize seaweed as a food source.

Recently, *Microbulbifer* species strain 6532A was isolated from seaweed along the coast of Japan, by Nakamura and colleagues while looking for bacteria capable of degrading seaweed [15]. Four proteins were identified by zymogram analysis as capable of degrading alginate. The smallest of these proteins, at 38kDa, is likely represented by a sequence in the genome that was identified as a putative alginate lyase, *algMsp*. In this report, we express, purify, and characterize the alginate lyase AlgMsp.

## Methods and Materials

Certain commercial materials and equipment are identified in this paper in order to adequately specify the experimental procedure. Such identification implies neither recommendation or endorsement by the National Institute of Standards and Technology nor that the material or equipment identified is the best available for the purpose.

### Chemicals

All restriction enzymes, T4 DNA ligase, and Phusion DNA polymerase were purchased from New England Biolabs (Ipswich, MA). Alginic acid, sodium salt, low viscosity (cat. no. 154725), was from MP Biomedicals LLC (Solon, OH). Carbazole, (3,5)-dinitrosalicylic acid, and all standard chemicals were purchased from Fisher Scientific (Pittsburgh, PA). Polymannuronic acid and polyguluronic acid were purchased from Carbosynth US LLC (San Diego, CA).

### Cloning, expression, and purification of alginate lyase AlgMsp

Lyase *algMsp*, GenBank Gene accession AB603802, was chosen by sequence analysis as one of several candidate alginate lyases for characterization. A codon-optimized clone was produced by GeneArt Inc. (San Francisco, CA), subcloned into pBAD24 using EcoRI and HindIII restriction sites, and sequences for a thrombin site and His6 tag were added before the stop codon. This added the following peptide to the end of AlgMsp: KLVPRGSRHHHHHH. The N-terminus of AlgMsp is predicted to have a signal peptide sequence by the SignalP4.1 program [16]. To improve expression, the first 30 codons of AlgMsp were replaced by a single ATG start codon. The pBAD24-AlgMsp clone was transformed into *E. coli* BL21(DE3) cells. An overnight starter culture was inoculated into 1.5 L of LB supplemented with 100 µg/ml ampicillin and grown in a 37°C shaking incubator at 180 rpm until the cells grew to mid-log phase. The culture was then put on ice and induced with 0.05% L-arabinose. Protein expression was induced for a total of 16 hours at 18°C in the shaking incubator at 180 rpm. Cells were pelleted by centrifugation at 7000 rpm for 15 minutes at 4°C, resuspended in 1/10^th^ volume QNL buffer (Qiagen Native Lysis buffer: 50 mM NaH_2_PO_4_, 300 mM NaCl, 10 mM imidazole, pH 8), and lysed by sonication on ice using a Branson sonifier set at 30% duty cycle and 60% power output for 10 minutes. The lysate was cleared by centrifugation at 22000×g for 1 hour at 4°C, and the supernatant was run over a 25 ml nickel column for purification by the His6 tag. The protein was eluted from the column with 50 ml 250 mM imidazole in PBS, pH 8, supplemented with 150 mM NaCl. The eluted protein was concentrated down to 10 ml and further purified by S-200 gel filtration chromatography in PBS, pH 8, supplemented with 200 mM NaCl. Protein was eluted with a flow rate of 2 ml/minute for 400 ml, and 5 ml fractions were collected. Pure fractions were dialyzed against 20 mM Tris, 200 mM NaCl, pH 8, for enzyme activity testing. AlgMsp was re-dialyzed into other buffers as needed. Purified protein was stored at 4°C. Protein concentrations were determined by Bradford assay, using BSA as the standard. Protein aliquots were run on a 10% SDS-PAGE gel to assess solubility and purity.

### Alginate lyase activity assays

Activity of the alginate lyase was determined by the increase in absorbance at 235 nm due to the formation of a carbon-carbon double bond at the end of the product generated from lyase-mediated cleavage of alginate [17]. For determining optimal pH and NaCl conditions at 25°C, 1 µg/ml lyase was used with 1 mg/ml alginate. Typically, AlgMsp lyase was incubated with alginate in 20 mM Tris, 200 mM NaCl, pH 8 buffer in a 200 µL reaction volume using a UV-transparent 96 well quartz plate (Molecular Devices, Sunnyvale, CA). Alginate was also prepared in 20 mM boric acid-phosphoric acid (BP) buffer (pH 4 to pH 10) and in 20 mM Tris (pH 7 to pH 9) for determining optimal pH. Measurements were taken using a SpectraMax M5 plate reader (Molecular Devices, Sunnyvale, CA) at 25°C. An absorbance reading at 235 nm was taken every 30 seconds for a total of 10 minutes to determine initial velocity for the reaction. Values were normalized to the maximal value. To determine the effects of cations on alginate lyase activity, AlgMsp was treated with 12.5 mM EDTA, pH 8 for 30 minutes, followed by EDTA removal by dialysis. Both EDTA-treated and untreated AlgMsp, 5 µg/ml, were incubated with 0.1 mM-10 mM cations for a total of 30 minutes. The alginate, comprised of the same cation and molar concentration, was then added to assay activity at 25°C. Final assay concentrations were 0.25 µg/ml AlgMsp, 3.6 mg/ml alginate, 20 mM Tris, 200 mM NaCl, pH 8 plus 0.1 mM-10 mM cation. Activity was determined by initial velocity as above.

Due to the limitations of the plate reader, temperature related assays were done as end-point runs. For determining the optimal reaction temperature, 200 µL reactions were done in thin-walled PCR tubes using a thermocycler with the lid heater off. The substrate was first pre-warmed for 3 minutes at various temperatures, followed by the addition of the enzyme. To accommodate increased activity of AlgMsp at specific temperatures tested, reactions conditions for this particular assay involved measuring activity from 0.25 µg/ml AlgMsp, 3.8 mg/ml alginate, 20 mM Tris, 200 mM NaCl, pH 8. AlgMsp activity was tested at temperatures from 10°C to 70°C. After incubating the samples for 10 minutes at the target temperature, the reaction was terminated by incubating the samples at 95°C for 10 minutes. Control reactions were run in parallel without enzyme. Aliquots of 180 µL were transferred to the wells of a clear plastic plate, and enzyme activity was determined as the difference in absorbance at 235 nm between the reactions with enzyme and the control reactions without enzyme. For examining the thermostability of AlgMsp activity, aliquots of 5 µg/ml AlgMsp, 20 mM Tris, 200 mM NaCl, pH 8, in eppendorf tubes were incubated for 24 hours in heatblocks set to 25°C, 37°C, and 50°C. Samples of AlgMsp lyase were taken at various time points, added to substrate, and assayed at the target temperature as above. All experiments were run at least three times with triplicate data points within each run.

### Circular dichroism (CD) spectropolarimetry

CD experiments for AlgMsp were performed on a Chirascan CD Spectrometer (Applied Photophysics) equipped with a thermoelectrically controlled cell holder. CD spectra were obtained in the far-UV range (190 nm–260 nm) in 1 nm steps using a 1 mm path length quartz cuvette with 5 second signal averaging per data point. Spectra were collected in triplicate and the Pro-Data software (Applied Photophysics) was used for averaging, baseline subtraction, smoothing and conversion to mean residue ellipticity (MRE). Secondary structure prediction was performed using the Provencher and Glockner method [18] located at the DICHROWEB [19] website. Melting experiments were performed by heating AlgMsp at a 0.1 mg/ml concentration in 20 mM sodium phosphate buffer pH 8 from 20°C to 95°C using a 1°C/min heating rate. MRE was monitored at 218 nm in a 1 mm path length quartz cuvette at 0.5°C steps with 5 second signal averaging per data point. Melting experiments were repeated to ensure reproducibility of the results.

### Enzyme kinetics assays

Reactions in 200 µl volumes were set up in triplicate in a 96 well quartz plate. Alginate, polyG, and polyM substrates were in 20 mM Tris, 200 mM NaCl, pH 8 buffer. Substrate concentrations ranging from 4 mg/ml to 0.0625 mg/ml using two-fold serial dilutions were assayed. The quartz plate and substrate were equilibrated to 50°C, followed by the addition of the enzyme to obtain a final protein concentration of 0.25 µg/ml. Initial velocities were determined from each reaction over a ten minute run. To determine enzyme kinetics, the averaged initial velocities in milli-absorbance units (mAU) at 235 nm per minute versus the substrate concentrations were determined. As alginate is a polymer of variable length consisting of random combinations of mannuronic acid and guluronic acid residues, and as both have the same molecular weight (MW), substrate molarity was calculated using the MW of 176 g/mol for each monomer of uronic acid in the polymer (i.e. 194 g/mol monomer MW – 18 g/mol for the loss of H_2_O during polymerization). Therefore, 4 mg/ml alginate is 22.7 mM monomer of uronic acid. Product concentrations were determined from the increase in absorbance at 235 nm using the extinction coefficient of 6150 M^−1^ cm^−1^ [20]–[22]. Velocity (V) at the tested substrate concentration was calculated as follows: V (mol/s)  =  (milliAU/min × min/60 sec × AU/1000milliAU × 1 cm)/(6150 M^−1^ cm^−1^) × (2 ×10^−4^ liters). Substrate molar concentrations and their associated velocity values were input in the Hyper32 program, http://homepage.ntlworld.com/john.easterby/hyper32.html [23] for calculating the maximal velocity, V_max_, and the Michaelis-Menten constant, K_m,_ for each substrate using hyperbolic regression analysis. The turnover number, k_cat_, for AlgMsp was calculated as follows: k_cat_ (s^−1^)  =  V_max_/E, where E is the mols of AlgMsp in the assay. Recombinant AlgMsp has a MW of 36,813 g/mol, therefore, at an enzyme concentration of 0.25 µg/ml, AlgMsp is equivalent to 6.79 nM, which is 1.36 pmol per 200 µl reaction.

### Reducing sugars assay

The DNS reagent consists of 3,5-dinitrosalicylic acid, 20 mg/ml, in 0.7 M NaOH. When heated, the DNS reacts with reducing sugars to generate products that absorb at 535 nm. The assay consists of mixing equal volumes of sample and DNS reagent, boiling for 5 minutes, cooling to room temperature, and then measuring the absorbance [24]. For high numbers of samples, a 96-well plate version was used. This consisted of pipetting 90 µl sample with 90 µl DNS reagent in each sample well of a 96-well PCR plate. The plate was incubated in a thermocycler at 95°C for 5 minutes and then cooled to 4°C. An aliquot of 150 µl from each sample was transferred to a 96-well clear plastic plate at room temperature. The absorbance of each sample well was then measured at 535 nm. Standard curves were made using glucose and alginate covering concentrations ranging from 0.125 mg/ml–1 mg/ml.

### Uronic acid assay

The uronic acid assay [25], [26], which involves carbazole reacting with uronic acid to generate products that absorb at 530 nm, was set up in a 96-well PCR plate. First, 12 µl of sample, 4 µl 0.1% carbazole (w/v in ethanol), and 133 µl 0.1 M borate (in concentrated sulfuric acid) were added to each well. The plate was sealed with adhesive film, incubated at 55°C for 30 minutes, and then cooled to 25°C for 2 minutes in a thermocycler. Aliquots of 120 µl were transferred to a 96-well clear plastic plate and the absorbance read at 530 nm. Standard curves were made using glucose and alginate covering concentrations ranging from 0.125 to 1 mg/ml.

### Analysis of reaction products by FPLC and mass spectrometry

In order to facilitate mass spectrometry analysis, alginate at 4 mg/ml in 5 mM NH_4_OAc, pH 7.4, was digested by 0.25 µg/ml of AlgMsp at 37°C for 18 hours. The reaction products, 0.5 ml, were separated by fast protein liquid chromatography (FPLC) using the Superdex Peptide HR10/30 gel filtration column (GE Life Sciences, Piscataway, NJ) in 20 mM NH_4_OAc, pH 7.4, at a flow-rate of 0.5 ml/min while collecting 0.25 ml fractions. The UV absorbance of unsaturated oligo-uronates has a maximum absorbance at 235 nm, but it is still detectable at 254 nm at a lower intensity allowing us to utilize our FPLC UV-detector for real time observation of the products. These UV peaks were confirmed by transferring FPLC fraction aliquots, 150 µl each, to a 96-well quartz plate and re-testing UV absorbance at 235 nm for unsaturated uronic acids. Fraction aliquots were further tested for total uronic acid content, saturated and unsaturated, by the carbazole assay, and for the presence of reducing sugars by the DNS assay. From separate FPLC gel filtration runs, four fractions representing the FPLC peaks were analyzed in the ion trap of a Thermo LTQ Orbitrap Discovery (San Jose, CA) mass spectrometer operating in negative ion mode. Samples for analysis were prepared by diluting each gel filtration fraction by tenfold in water. Samples were introduced by direct infusion (30 µl/min) into the electrospray ionization source (ESI), and mass spectra (MS) were collected. To help elucidate the structure of the ESI-MS peaks, MS/MS spectra were collected concurrently by isolating specific *m/z* anions, fragmenting the anions by subjecting them to 35 eV collisions with helium, and recording the product ion spectra with the ion trap. Purging the electrospray source with 0.25 ml water, and, as needed, 0.25 ml methanol, eliminated measureable carryover signal between samples.

## Results

### Cloning and expression

**Figure 1:**
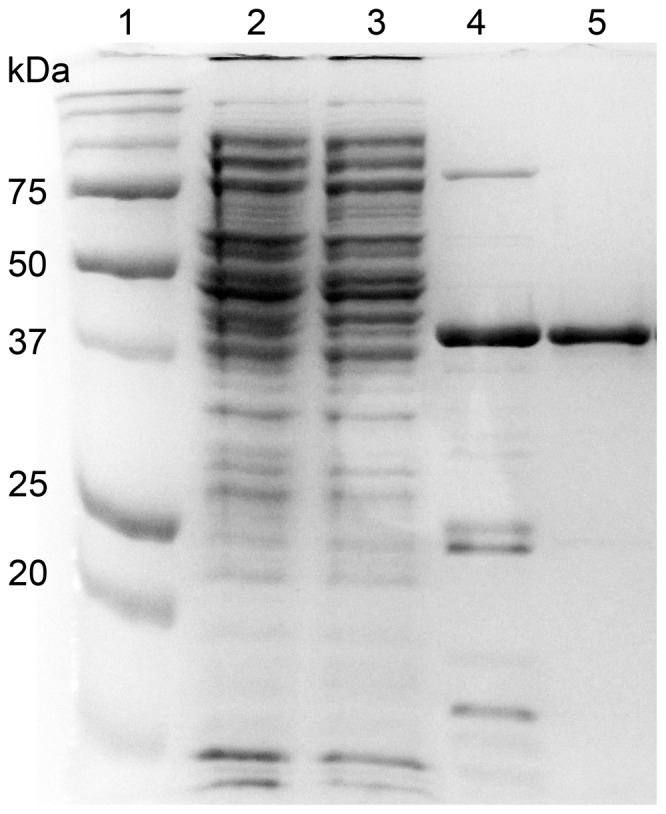
SDS-PAGE analysis of recombinant AlgMsp expression and purification. Lane 1 is the Precision Plus protein standard (Bio-Rad). Lane 2 is the cell lysate. Lane 3 is the flow-through from the nickel column. Lane 4 is the elution from the nickel column. Lane 5 is the elution from the S-200 gel filtration column.

A synthetic *algMsp* gene was produced by GeneArt Inc., and subcloned into pBAD24. The native AlgMsp protein is 358 amino acids long. Early attempts to express the His-tagged full-length AlgMsp protein were not successful. Analysis of its sequence using the SignalP4.1 program [16] suggested the presence of a signal peptide. Deletion of the predicted signal peptide sequence increased expression of soluble protein. The expressed recombinant protein, including a C-terminal thrombin site and His6 tag, is 343 amino acids long, with a predicted molecular weight of 37 kDa. The enzyme was purified using a nickel column and then by gel filtration chromatography. SDS-PAGE confirmed AlgMsp achieved ≥ 95% purity at the end of the purification process (figure 1, lane 5). The purified protein runs at about 39 kDa; close to the predicted size for AlgMsp.

### Effect of pH and NaCl on activity

**Figure 2:**
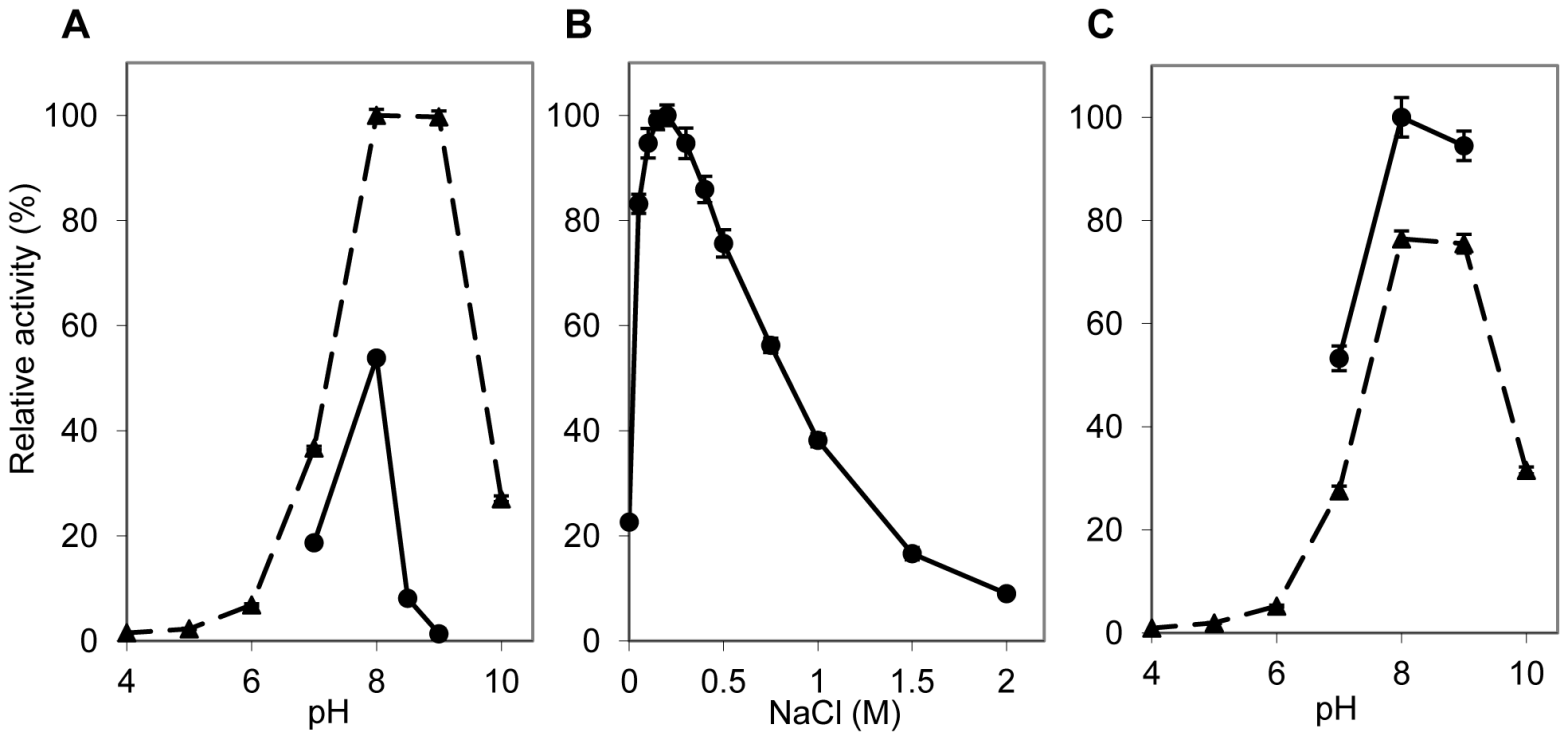
Effects of pH and NaCl on alginate lyase activity of AlgMsp. (A) Effect of pH in the absence of NaCl on activity in 20 mM Tris (circles) or BP buffer (triangles). (B) Effect of NaCl on activity in 20 mM Tris buffer pH 8. (C) Effect of pH on activity in the presence of 200 mM NaCl in 20 mM Tris (circles) or BP buffer (triangles). Activity determined by the initial velocity of increase in absorbance at 235 nm over a 10 minute incubation at 25°C. Activity was normalized to 100% for the most active sample.

AlgMsp alginate lyase activity was tested against commercially available seaweed alginate, and characterized for the effects of pH and NaCl on its activity. Initial testing to elucidate the optimal pH for AlgMsp in the absence of NaCl showed a preference for pH 8 in Tris buffer, but a broader range of pH 8 to pH 9 in boric acid-phosphoric acid (BP) buffer (figure 2a). In the absence of NaCl, activity was greater in the BP buffer than in the Tris buffer.

Next, the salt-dependence of AlgMsp was determined using NaCl concentrations ranging from 0 M to 2 M in Tris buffer, pH 8. The optimal NaCl concentration for activity was 200 mM, with >80% activity observed between 100 mM to 400 mM NaCl (figure 2b). The enzyme retained 10% activity at 2 M NaCl and >20% activity in the absence of NaCl. Examining the optimal pH in 200 mM NaCl showed greatest activity in Tris buffer at pH 8 (figure 2c). In the presence of salt, the overall activity of AlgMsp was lower in BP buffer compared to the activity exhibited by the enzyme in Tris buffer. HEPES and bicine buffers also showed peak AlgMsp lyase activity at pH 8, but at lower activity levels relative to that seen in Tris buffer (data not shown).

### Effect of temperature on activity

**Figure 3:**
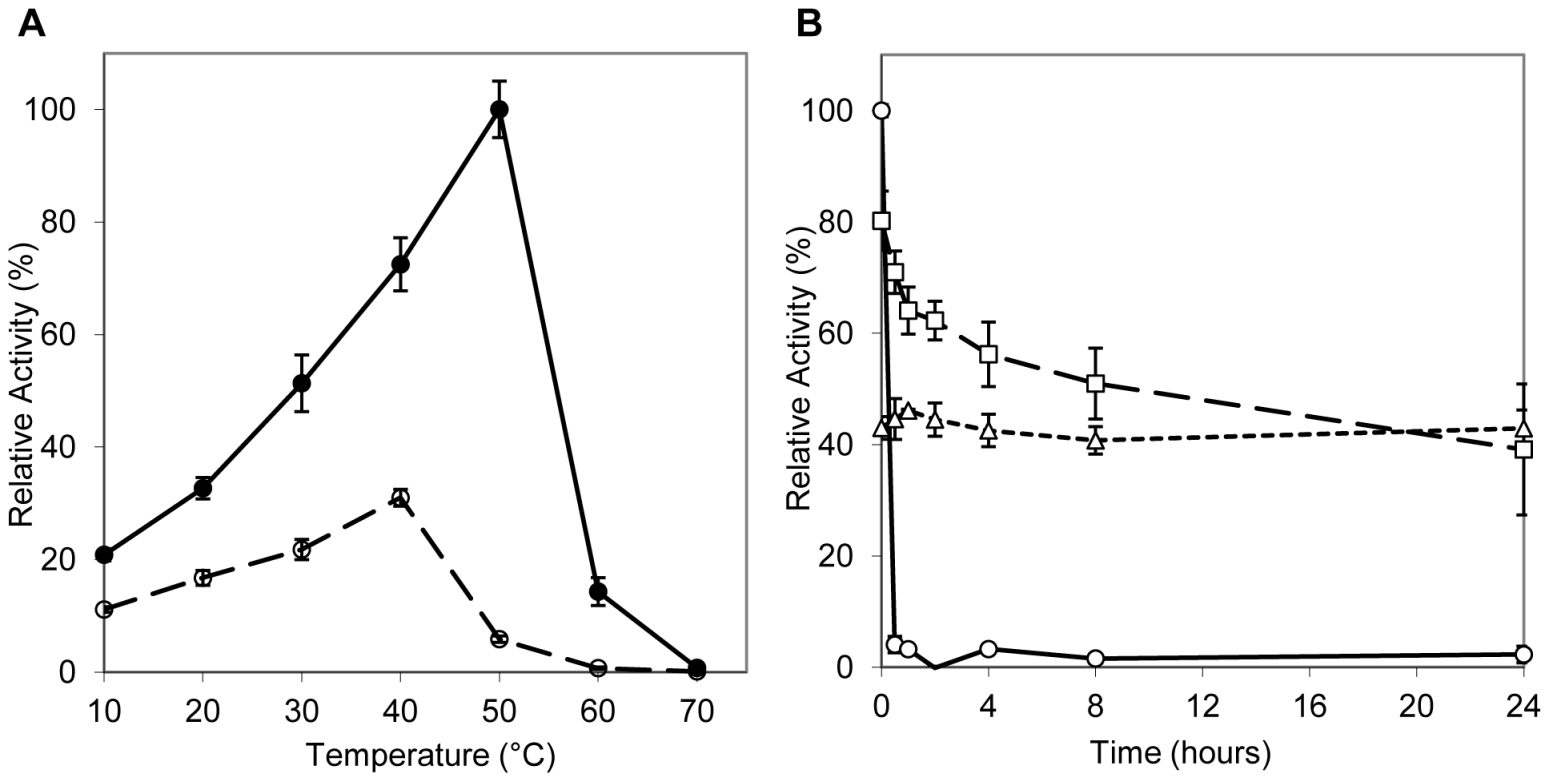
Effect of temperature on alginate lyase activity and stability of activity. (A) Effect of temperature on activity with (filled circles) and without NaCl (open circles). Activity was determined, after 10 minutes at the target temperature, by the increase in absorbance at 235 nm over the absorbance of the control reaction without enzyme. (B) Thermostability of alginate lyase activity. AlgMsp was incubated at 50°C (circles), 37°C (squares), and 25°C (triangles) for 24 hours in 20 mM Tris, 200 mM NaCl, pH 8. Samples were tested at 0, 0.5, 1, 2, 4, 8, and 24 hours for residual activity as above. Activity was normalized to 100% for the most active sample.

Temperature effects on AlgMsp lyase activity and stability were examined from 10°C to 70°C. In the presence of salt, the optimum temperature for enzyme activity was observed at 50°C (figure 3a, filled circles). A dramatic reduction in activity, down by 86%, was observed when AlgMsp was introduced to 60°C, and all activity was absent at 70°C. In the absence of salt, activity peaked at 40°C (figure 3a, open circles). Thermostability of AlgMsp was tested in the presence of salt at room temperature (25°C), human body temperature (37°C) and peak activity temperature (50°C). In this study, the enzyme was incubated at the target temperatures for a total of 24 hours, with the residual activity being assayed at those temperatures at various time points. At 50°C, which was previously determined to be the optimal temperature for activity, almost all activity was lost after 30 minutes (figure 3b, circles). At 37°C, AlgMsp residual activity gradually declined to half its initial level over the duration of the 24 hour incubation (figure 3b, squares). Finally, the activity of AlgMsp at 25°C remained relatively unchanged over 24 hours (figure 3b, triangles). Therefore, while 50°C is the optimal temperature for AlgMsp activity, this temperature is not practical when prolonged use of the enzyme is required.

### Circular dichroism analysis

**Figure 4:**
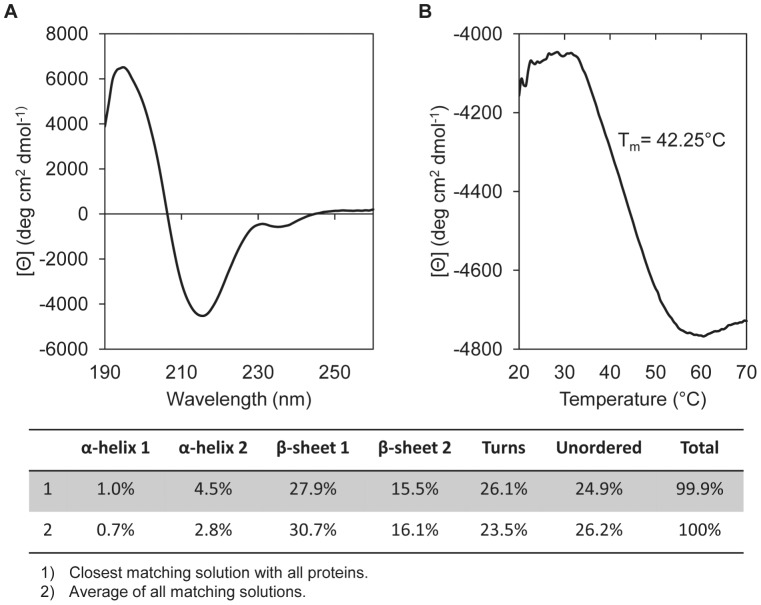
Circular dichroism analysis of stability and structure of AlgMsp. (A) Far-UV CD analysis of AlgMsp structure, representative graph displaying the calculated secondary structure composition. (B) CD-melting curve of AlgMsp. The enzyme was heated at 1°C/min in 20 mM sodium phosphate buffer pH 8 at a protein concentration of 0.1 mg/ml.

Circular dichroism (CD) analysis of AlgMsp was carried out in sodium phosphate buffer, pH 8. CD was first used to determine the protein secondary structure composition of AlgMsp. The far-UV CD spectra for AlgMsp indicates that it consists predominantly of β-sheets (>40%) and turns (∼25%) with little α-helix content (<6%) (figure 4a). This is in agreement with the secondary structure composition of the native protein predicted by the JPred3 and PSIPRED programs [27], [28], which calculated high β-sheet content (∼29%) with a small number of α-helices (3.5%). CD melting experiments, which monitored the integrity of β-sheet secondary structure over a range of temperatures, revealed that AlgMsp has a T_m_ of 42.25°C (figure 4b). This is in agreement with the loss of activity above 40°C seen for AlgMsp in the absence of salt (figure 3a, open circles). Moreover, AlgMsp displayed a thermal transition at 42°C when analyzed by differential scanning calorimetry (DSC), further confirming the CD results (data not shown).

### Divalent cation effects on activity

The activity of AlgMsp pre-treated with EDTA to remove bound ions, was investigated in the presence of several different divalent cations (table 1). Addition of high levels of calcium or other divalent cations to alginate results in formation of a gel [1]. As a result, divalent cations were used at concentrations that did not promote alginate gel formation. Calcium at a 1 mM concentration reduced AlgMsp activity 11% whereas magnesium at 1 mM and 10 mM concentrations reduced enzyme activity by 17% and 43%, respectively. In all cases, the cations tested proved inhibitory to AlgMsp activity. No significant differences between EDTA-pre-treated and untreated AlgMsp activity were seen (data not shown).

**Table 1:** Effects of divalent cations on AlgMsp activity.

Compound	AlgMsp Activity*
Control	100±4.9
10 mM MgCl_2_	54±3.8
1 mM MgCl_2_	78±1.5
1 mM CaCl_2_	86±6.3
0.1 mM CaCl_2_	93±2.0
10 mM MnCl_2_	40±2.1
1 mM MnCl_2_	63±3.2
10 mM NiCl_2_	3±0.7
1 mM NiCl_2_	13±0.2
1 mM Zn(OAc)_2_	10±3.1
1 mM CoCl_2_	25±0.7
1 mM CuSO_4_	22±3.9
1 mM FeCl_2_	−1±2.1

^*^Values expressed as relative activity (%) ± standard deviation.

### Enzyme kinetics

Testing AlgMsp lyase with varying concentrations of substrates revealed a lower K_m_, or higher binding affinity, for polyG than for polyM or alginate (table 2). Interestingly, the rate of catalysis, k_cat_, was lower for polyG than for polyM or alginate. This suggests that, while AlgMsp prefers to bind polyG, the lyase will cleave polyM more rapidly. Furthermore, the K_m_ for alginate is 3.4 mM, which is in between the values obtained for polyG and polyM substrates. However, the k_cat_ from alginate as a substrate was closer to that of polyM. This particular alginate is from *Macrocystis pyrifera* and has been reported to be 61% mannuronic acid and 39% guluronic acid [29]. Therefore, it is not unexpected that the turnover rates of alginate and polyM by AlgMsp are more similar than that of alginate and polyG.

**Table 2:** Kinetics of AlgMsp.

Substrate	V_max_ (pmols/sec)	K_m_ (mM)	k_cat_ (s^−1^)	k_cat_/K_m_ (M^−1^ s^−1^)
alginate	57±5	3.4±0.9	42	12300
polyG	35±2	1.8±0.4	26	14200
polyM	63±8	6.8±2.1	46	6840

Values ± standard error.

### Enzyme products

**Figure 5:**
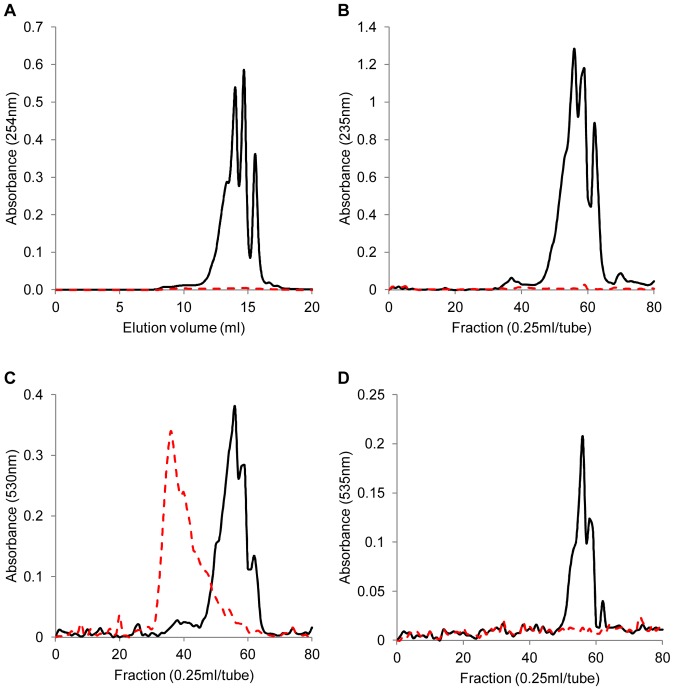
FPLC fractionation and analysis of products from AlgMsp-cleavage of alginate. AlgMsp-cut and uncut alginate are represented by a solid black line and a dashed red line respectively in panels A to D. (A) Real-time FPLC UV-detector trace from absorbance at 254 nm of products eluting from the Superdex Peptide HR10/30 column. (B) Testing of 0.25 ml FPLC fractions for unsaturated uronates by absorbance at 235 nm. (C) Testing of 0.25 ml FPLC fractions for all uronic acids by the carbazole assay with an increase in absorbance at 530 nm. (D) Testing of 0.25 ml FPLC fractions for reducing sugars by the DNS assay with an increase in absorbance at 535 nm.

The digested oligo-uronate products resulting from an 18 hour incubation of AlgMsp with alginate were fractionated by gel filtration chromatography for further analysis. ESI-MS is sensitive to the presence of sodium. Therefore, the enzyme reactions and FPLC elution were done in ammonium acetate to allow for direct measurements of the peak fractions by mass spectrometry. The absorbance curve from the double bond created in the cleaved alginate by the lyase has a peak absorbance at 235 nm. The span of the absorbance peak allows the digested products to be measured by the 254 nm filter on the FPLC UV-detector. Conversely, uncut alginate does not absorb at 235 nm or 254 nm. The UV absorbance chromatogram for AlgMsp-cut alginate shows 4 peaks (figure 5a, black trace), whereas alginate alone had no peaks (figure 5a, red dashes). The 0.25 ml FPLC fractions were collected and tested for unsaturated uronates, all uronates, and reducing sugars. Unsaturated uronic acid maximally absorbs UV at 235 nm. The FPLC fractions from AlgMsp-cut alginate show the same set of peaks from absorbance at 235 nm as those from the FPLC detector at 254 nm (figure 5b, black trace). Thus, the fractions accurately depict that same data. Carbazole reacts with uronic acids to generate a product that absorbs at 530 nm. The carbazole assay performed on the FPLC fractions from AlgMsp-cut alginate developed a similar pattern of peaks as was detected by UV absorbance (figure 5c, black trace). This finding confirms that the FPLC peaks contain uronic acid. The FPLC run with uncut alginate also illustrates the presence of uronic acids at earlier elution volumes (figure 5c, red dashes). The uncut alginate starts eluting in the void volume for the column (8 ml by elution of BSA, data not shown), which is expected as uncut alginate should be larger and elute sooner than alginate digested by AlgMsp. The DNS assay for reducing sugars from the fractions of the AlgMsp-cut alginate also showed the similar pattern of peaks seen during FPLC (figure 5d, black trace). No reducing sugars were detected for the untreated alginate (figure 5d, red dashes). Therefore, the reducing sugars present are due to the action of AlgMsp.

Fractions representing the peaks seen in figure 5 were examined by electrospray ionization mass spectrometry (ESI-MS) and by ESI-MS/MS to determine the composition of the AlgMsp-digested alginate. Alginate is a linear polymer consisting of two monomers, guluronic acid and mannuronic acid, with the same molecular weights. AlgMsp cleavage of alginate generates two oligo-uronates. From the cleavage site, one oligo-uronate receives a reducing sugar at its new end, and the other oligo-uronate receives a non-reducing unsaturated uronate at its new end. The oligo-uronates are commonly described by their degree of polymerization (DP*x*). Oligo-uronates with the unsaturated terminal uronate are denoted in this study as ΔDP*x*, and such a trimer would be ΔDP3. Rare saturated oligo-uronates arise from the original terminus of the alginate polymer, and likely more saturated ends are artificially generated from the commercial processing of the seaweed alginate prior to its use as substrate in an enzymatic assay. A saturated dimer would be DP2 and a saturated trimer would be DP3.

**Figure 6:**
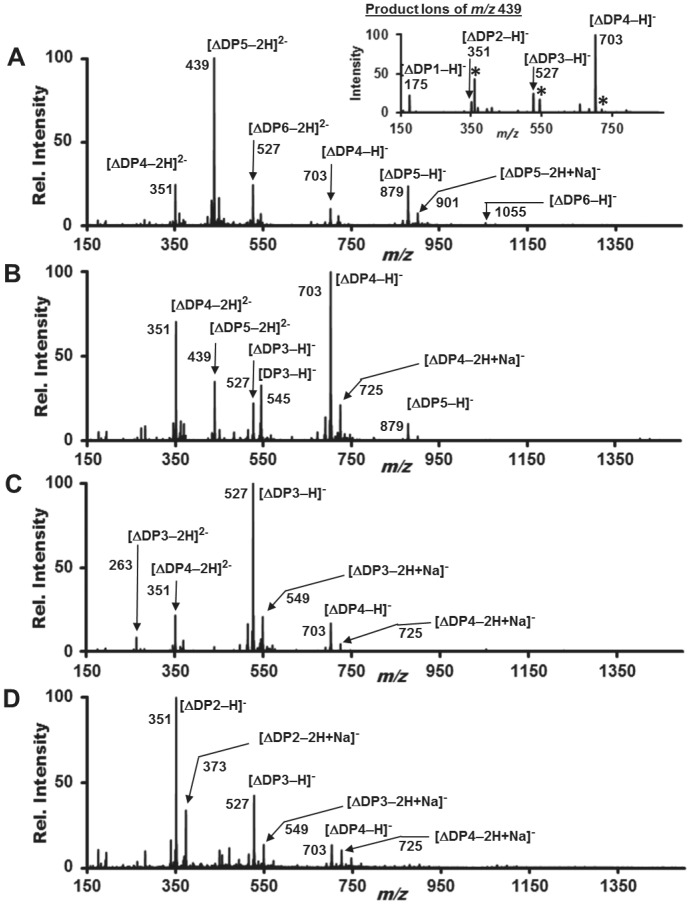
ESI-mass spectrometry analyses of peak fractions from FPLC separation of the oligo-uronates from AlgMsp digestion of alginate. DPx is degree of polymerization. DPx and ΔDPx represent saturated and unsaturated oligo-uronates, respectively. (A) Mass spectrum of FPLC fraction 1, the 13.4 ml elution peak, showing predominance of ΔDP5 eluent. Inset: MS/MS product ion spectrum of *m/z* 439 peak, showing the methodology used to elucidate the structure of all observed ESI-MS anion peaks. The *m/z* 703 and *m/z* 721 peaks are indicative of the double charge of the precursor anion, *m/z* 439. The annotated peak series, {[ΔDP*x*-1H]^−^, *x* = 1–4}, and the peaks denoted with asterisks ([DP4-2H]^2−^ (*m/z* 360), {[DP3-H]^−^ (*m/z* 545), and {[DP4-H]^−^ (*m/z* 721), confirm that the composition of the *m/z* 439 precursor anion, is consistent with [ΔDP5-2H]^2−^. (B) Mass spectrum of FPLC fraction 2, the 14.0 ml elution peak, showing predominance of ΔDP4 eluent. (C) Mass spectrum of FPLC fraction 3, the 14.7 ml elution peak, showing predominance of ΔDP3 eluent. (D) Mass spectrum of FPLC fraction 4, the 15.6 ml elution peak, showing predominance of ΔDP2 eluent.

In figure 6, panels 6a to 6d represent the mass spectrometry analyses of the FPLC peaks at 13.4 ml (fraction 1), 14.0 ml (fraction2), 14.7 ml (fraction 3), and 15.6 ml (fraction 4), respectively. These data are plotted as relative ion intensity vs. ion mass/charge ratio (*m/z*). In figure 6a, analysis of fraction 1, the base peak, *m/z* 439, and the *m/z* 879 peak provide evidence that the solution contains ΔDP5 predominately. The less intense *m/z* 1055 and *m/z* 527 peaks indicate that the solution also contains some ΔDP6. The less intense *m/z* 351 and *m/z* 703 peaks suggest that the sample contains some ΔDP4. The signal arises from negative ions, typically, of compositions consistent with oligo-uronate molecules that have lost one or more protons. The calculated MW of ΔDP5 is 880 Da, but due to the loss of a proton, the single charge anion, [ΔDP5-H]^−^, is observed at 879 *m/z*. In the case of the *m/z* 439 peak, anionic species form from ΔDP5 through the loss of two protons during the electrospray ionization process, resulting in the mass/charge ratio of (880–2H)/2charges  =  439 *m/z*.

The data set does not support the alternate interpretation that would account for the ESI-MS spectrum by attributing the eluent to ΔDP6 solely. In this interpretation the [ΔDP5-*x*H]*^x^* ^−^ and [ΔDP4-*x*H]*^x^* ^−^ (where *x* = 1,2) peaks must arise through neutral loss of one or two oligo-uronates from [ΔDP6-*x*H]*^x^* ^−^ precursor anions, induced either by collisions in the ESI source or via thermal decomposition. When such in-source fragmentation processes are significant, the product ion intensity ratios can vary with the electrical potential or temperature within the ESI source. These effects were not observed. Peak intensity ratios in the ESI-MS spectra remained constant as the tube lens in the ESI source was adjusted from 0 V to–140 V and the capillary temperature was varied from 40°C to 275°C. Thus, while some activity of neutral loss dissociation channels cannot be ruled out, we conclude that such activity is minimal.

The composition of each anion observed in the ESI-MS spectra of figure 6 was confirmed by MS/MS experiments. The insert of figure 6a shows the product ion spectrum produced by the MS/MS experiment that fragmented *m/z* 439 precursor anions. During each MS/MS experiment the precursor anion was isolated in the ion trap, subjected to 35 eV collisions with helium, and the product ion spectrum was recorded. As shown in the insert of figure 6a, the prominent *m/z* 703 product ion peak and the much less intense *m/z* 721 peak, which both reside at higher *m/z* than the precursor anion, are direct evidence that the precursor contained double charge. The sequence of *m/z* 703, *m/z* 527, *m/z* 351, and *m/z* 175 peaks in the product ion spectrum arises from loss of {[ΔDP*x-*H]^−^, *x* = 1–4}, respectively, from *m/z* 439 precursor double charge anions. Our data does not distinguish between loss through a single reaction process or through combinations of charge fission and neutral loss steps. Additionally, the MS/MS spectrum of *m/z* 439 precursor anion exhibits less intense peaks, denoted by asterisks, produced by the loss of the unsaturated terminus of ΔDP5 to yield {[DP4-2H]^2−^ (*m/z* 360), {[DP3-H]^−^ (*m/z* 545), and {[DP4-H]^−^ (*m/z* 721). Taken together, these data confirm that the *m/z* 439 precursor evidences [ΔDP5-2H]^2−^, which originates from ΔDP5 eluent. Furthermore, peaks from [ΔDP*x*-2H+Na]^−^ (*x* = 2–6) species in the ESI-MS spectra of Figure 6 confirm the corresponding assignments of ΔDP*x* among the eluent fractions.

Figure 6b shows the MS spectra of the next FPLC peak, fraction 2. The base peak is *m/z* 703 ([ΔDP4-H]^−^), and the second most intense peak is *m/z* 351 ([ΔDP4-2H]^2−^, indicating that the eluent fraction contains ΔDP4 predominately. Weaker anion peaks evidence the presence of some ΔDP3 and ΔDP5. The MS spectra of fraction 3, in figure 6c, shows *m/z* 527 base peak ([ΔDP3-H]^−^), indicating that the eluent fraction contains ΔDP3 predominately. The less intense *m/z* 703 peak evidences the presence of some ΔDP4 in this sample. Peaks due to sodium ions bound to oligo-uronate anions also evidence the presence of ΔDP4 and ΔDP3. Finally, in figure 6d, for fraction 4, the base peak is *m/z* 351 ([ΔDP2-H]^−^), which represents ΔDP2. Less intense peaks from ΔDP3 (*m/z* 527) and ΔDP4 (*m/z* 703) indicate that these species are present in lower amounts. Thus, the major products from extended cleavage of alginate by AlgMsp are dimers (ΔDP2), trimers (ΔDP3), tetramers (ΔDP4), and pentamers (ΔDP5).

## Discussion

In this study, AlgMsp, an alginate lyase from the marine bacteria *Microbulbifer sp. 6532A*, was expressed and characterized. AlgMsp belongs to the alginate lyase 2 family, pfam08787, of the Conserved Domains database [30]. Members of pfam08787 have a predominantly beta sheet structure. This was confirmed by far-UV CD analysis of AlgMsp (figure 4a). AlgMsp is also defined as a member of the polysaccharide lyase family 7 (PL7), subfamily 5, by the Carbohydrate Active Enzymes database (http://www.cazy.org/) [31]. The CAZY website lists the PL7 subfamily 5 group as currently having 50 members, of which seven of these alginate lyases are listed as characterized. Three of these alginate lyases, that have published data for comparison, are as follows: A1m (cited as AlgL in GenBank, accession number BAG70358) from *Agarivorans sp*. JAM-A1m [32], AlyPI (GenBank ACM89454) from *Pseudoalteromonas sp*. CY24 [33], and AlyDW11 (GenBank AEO50363) from an uncultured bacterium from the gut of the sea snail, abalone [34]. The closest matches to AlgMsp by protein BLAST [35] are with sequence-defined alginate lyases from *Microbulbifer agarilyticus*, (GenBank WP_010131148) with 79% identity, and from *Saccharophagus degradans* 2-40, (GenBank YP_527950) with 74% identity. There is a poly-serine region near the N-terminus of AlgMsp that is shared with the alginate lyase from *Microbulbifer agarilyticus*. This poly-serine region is not necessary for catalytic activity, as a mutant AlgMsp with that region deleted still had activity (data not shown).

Most examined bacterial alginate lyases have optimal activity in the range of pH 7 to pH 8.5 [3]. The optimal pH for lyase A1m is pH 9, while the optimum pH for lyases AlyPI and AlyDW is pH 7. AlgMsp optimal pH, at pH 8, is between those values (figure 2). Depending on the buffer and salt composition, the pH range at which AlgMsp displays activity can range from pH 6 to pH 10. This pH range is similar to that seen for the 3 other PL7 subfamily 5 lyases. Optimal salt concentrations for enzyme activity can vary considerably. AlgMsp is most active at 200 mM NaCl, whereas for A1m it is 600 mM NaCl [32] and for AlyPI it is 100 mM NaCl [33]. AlgMsp shows the ability to retain activity over the broad range of 0 M to 2 M NaCl. AlyPI shows a similar degree of activity over most of that range, whereas A1m shows greater activity across a higher range of NaCl concentrations.

In optimal buffer conditions, the temperature at which AlgMsp activity is highest was 50°C (figure 3a, filled circles). Without NaCl, the optimal temperature was lowered to 40°C (figure 3a, open circles). As a comparison, the optimal temperature for A1m is 30°C [32], for AlyPI is 40°C [33], and for AlyDW is 45°C [34]. The activity displayed by AlgMsp at different temperatures is partly influenced by how stable the enzyme is at a particular temperature as well as the total time required to complete the assay. In figure 3b, the activity over time at different temperatures showed that while activity was greatest at 50°C, it did not last more than 30 minutes at 50°C. However, at 25°C, although the initial activity of AlgMsp was about 40% that of the initial activity at 50°C, the residual activity of AlgMsp remained stable for entire duration of the 24 hour experiment (figure 3b, triangles). Additionally, the CD melting experiments (figure 4b) and DSC analysis (data not shown) indicate that the T_m_ for AlgMsp is ∼42°C. Due to the predicted signal peptide in the native protein, AlgMsp is likely found in the periplasmic space and/or secreted into the environment to aid in the breakdown of seaweed alginate. In terms of long-term enzyme stability, the temperature of the localized environment occupied by the marine microbe will ensure the lyase remains functional for an extended period of time since the enzyme will most likely be operating at temperatures closer to 25°C than to 50°C. The biochemical characteristics of AlyDW, a lyase from the uncultured bacterium from a sea snail gut, seem to most closely resemble those of AlgMsp.

Enzyme activity is often influenced by the presence of divalent cations, either as necessary cofactors for activity or improving stability, or alternatively these cations can inhibit activity by binding to induce protein conformation alterations or competing away other necessary cofactors. Specifically, calcium and magnesium are often stimulatory or required for activity of alginate lyases [3]. The divalent cation-dependence of AlgMsp was investigated by first treating the enzyme with EDTA to chelate away divalent cations, followed by measuring the activity of the AlgMsp after the consequent addition of various cations. The activity of the lyase without EDTA treatment was also assayed. First, as seen in table 1, EDTA treatment did not alter activity of the enzyme. This suggests that AlgMsp does not require a divalent cation as a cofactor. Second, all cations tested inhibited AlgMsp activity to some degree. CaCl_2_ at 1 mM slightly reduced activity to 89%, whereas NiCl_2_ at 1 mM reduced activity to just 13%. No difference was seen between EDTA-treated and untreated AlgMsp tested with these cations, further suggesting that no cofactors are required for AlgMsp activity. This suggests that AlgMsp is more susceptible to conformation alterations and/or destabilization from cations binding. Other alginate lyases respond differently to cations. AlyDW is unaffected or weakly inhibited by most commonly tested divalent cations, although the enzyme appears to be stimulated by AgNO_3_ [34]. On the other hand, AlyPI is stimulated by Mn^2+^, Ca^2+^, and Fe^3+^, and weakly inhibited by Ni^2+^, Zn^2+^, and Mg^2+^ [33].

Enzyme kinetics of alginate lyases can be difficult since the substrate alginate is heterogenous in its composition as different seaweeds have different mannuronic to guluronic ratios. Additionally, within the polymer, there are polyG, polyM, and polyMG regions, and the frequency of these regions can differ for a particular seaweed source [1], [3]. Production of purified alginate results in a mix of different length polymers, with their average length being dictated by the particular production methodology utilized. Therefore, comparisons of kinetics between different alginate lyase publications should not be done rigidly. Nonetheless, the K_m_ of AlgMsp to alginate was calculated to be 3.4 mM (table 2), which is similar to many alginate lyases that likewise have K_m_ values in the low milli-molar range [2]. For example, another PL7 family member, AlyA1 from *Zobellia galactanivorans*, was tested against different seaweed alginates and shown to have K_m_ values ranging from 1.7 mM to 6.2 mM, with increased binding affinity exhibited towards alginate with higher guluronate content [36]. Another example is Aly-SJ02, an alginate lyase from *Pseudoalteromonas* sp. SM0524, a marine organism, which has a K_m_ of 6.1 mM for the alginate monomer [37]. For marine organisms that feed on seaweed, the low binding affinities of lyases is acceptable due to the high concentration of alginate in seaweed, which represents 17%–45% of the mass of dried brown seaweed [38].

There are some notable exceptions of alginate lyases with K_m_ values in the micro-molar range. These include the AlgL lyase from *Pseudomonas aeruginosa*, which has a K_m_ of 11 µM for alginate [20], and the *Sphingomonas* A1 lyase A1-II’ binds alginate with a K_m_ of 8.6 µM [39]. The crystal structure of A1-II’ is known and the active-site residues have been determined by mutagenesis studies. It is interesting to note that sequence alignment of AlgMsp to A1-II’ shows the amino acids of the active-site cleft structure are conserved between them, however, AlgMsp has multiple insertions of 8–18 amino acids between the active cleft pieces relative to A1-II’ (data not shown). While structural studies are needed on AlgMsp, it is likely that these additional residues could alter the positioning of substrate in the active-site, which may offer clues to the ∼1000 fold difference in K_m_ between the two enzymes.

Different alginate lyases can have different polyM, polyG, or even polyMG specificities [3]. Additionally, it is typical for alginate lyases to prefer one substrate, but still cleave the other substrate at a reduced rate. For example, Aly-SJ02, an alginate lyase from Pseudoalteromonas sp SM0524, cleaves both polyG and polyM with specific activity against polyG being 75% that against polyM [37], and AlyV5, an alginate lyase from Vibrio sp QY105, has 3 times more activity with polyG compared to polyM [40]. As seen in table 2, AlgMsp has a lower K_m_ value (higher affinity) for polyG than for polyM. This suggests that AlgMsp is more likely to bind polyG tracts in the alginate polymer. While the V_max_ of AlgMsp is greater for polyM than polyG, the efficiency of cleavage as determined by k_cat_/K_m_ ratio is two-fold better for polyG than polyM. AlgMsp is bifunctional in that it acts on both polyG and polyM, but has a preference for polyG.

The rate of catalysis for AlgMsp with alginate was 42 s^−1^. Few alginate lyases have k_cat_ values determined for them. AlyA1 from *Zobellia galactanivorans* has a reported k_cat_ of 19.5 s^−1^ for alginate (33.3% guluronate) [36]. Additionally, AlgL from *Pseudomonas aeruginosa* has a reported k_cat_ of 32 s^−1^ for alginate [20]. These rates are very close to that for AlgMsp, and suggests that its turnover rate is not uncommon among alginate lyases.

Cleavage of alginate by lyase generates unsaturated oligo-uronates. For AlgMsp, we were able to demonstrate that these oligo-uronates form distinct peaks during gel filtration chromatography (figure 5a, black trace). Additionally, these peaks represent the uronic acid content, both saturated and unsaturated, and the reducing sugars of the digested alginate (figure 5b–d). Using mass spectrometry, we were able to show that the major products of prolonged digest of alginate by AlgMsp were unsaturated oligo-uronate dimers, trimers, tetramers, and pentamers (ΔDP2-ΔDP5). No monomers were detected by chromatography. This suggests that even though the end products are separated in size by a single uronic acid monomer, this size distribution is not due to trimming of monomer from the ends of the polymer chains. If AlgMsp were an exo-lyase and was trimming away from the ends, one would expect a build-up of the oligo-representing the optimal bite size of the enzyme [41], [42]. This was not observed. Instead, AlgMsp generated a mix of oligo-uronates, ΔDP2-ΔDP5. It is more likely that this distribution of oligo-uronates is due to the lyase cutting the polymer internally, suggesting that AlgMsp acts as an endo-lyase.

Short oligo-uronates have been reported as improving root growth in rice and carrots [43]. AlgMsp could be potentially useful in production of short oligo-uronates for fertilizer as it produces oligos in the dimer to pentamer range. Additionally, it could be used to produce these short oligo-uronates from seaweed stock for biofuel production [8]. For both of these applications, the substrate, seaweed alginate, can be supplied at a high concentration. The longevity of AlgMsp activity at room temperature, and its persistence of activity across a broad range of pH environments and salt concentrations increase the feasibility of utilizing this particular lyase for industrial applications.
